# Descriptive feedback with targeted education to improve telephonic escalation of care: a simulation-based study

**DOI:** 10.1186/s12909-024-05260-1

**Published:** 2024-03-13

**Authors:** Aster Kuriakose, Subodhini Puhambugoda Arachchige, Theophilus I Emeto, Matthew I Hiskens, Gopakumar Hariharan

**Affiliations:** 1 Department of General Paediatrics, Mackay Base Hospital, 4740 Mackay, QLD Australia; 2 Public Health & Tropical Medicine, College of Public Health, Medical & Veterinary Sciences, James Cook University, 4811 Townsville, QLD Australia; 3 Mackay Institute of Research and Innovation, Mackay Base Hospital, 475 Bridge Road, 4740 Mackay, QLD Australia

**Keywords:** Communication, Training, Education, Simulation

## Abstract

**Background:**

Awareness of communication failures in healthcare has necessitated the implementation of standardized, validated handover tools such as Identification, Situation, Background, Assessment, Recommendation (ISBAR). Although educational sessions improve communication, the effectiveness of individualized care escalation communication training is unknown. The primary aim was to conduct a simulation-based study to assess individualized one-on-one communication training for junior medical doctors for improving care escalation in pediatric emergencies. The secondary aim was to assess the evaluation of the training.

**Methods:**

The prospective observational study assessed participants pre- and post-intervention. In Session One, participants presented a written case scenario telephonically to two senior pediatricians. Fifty participants were scored using an 18-item checklist based on the ISBAR tool and “free text” responses. Immediately following case presentations, participants completed individualized one-on-one 30-minute educational sessions regarding self-reflection, didactic teaching, and constructive feedback based on the ISBAR. Session Two included a second case presentation and reassessment. We conducted qualitative analysis of supervisor’s feedback on performance and trainee doctor’s evaluation of the training.

**Results:**

There was significant improvement in 8 of the 18 components of the ISBAR checklist. All elements of care escalation were significantly improved, and overall communication was higher post-intervention (*P < 0.001*); however, no improvement was noted in participants’ explorations of differential diagnoses (*P = 0.263*). The qualitative analysis identified themes of improved urgency in seeking senior support and conversational clarity from supervisors, and improved intervention quality and self-confidence from participants.

**Conclusions:**

Individualized communication training may improve pediatric emergency care escalation and communication among junior doctors.

**Supplementary Information:**

The online version contains supplementary material available at 10.1186/s12909-024-05260-1.

## Background

Early recognition of patient deterioration and prompt responses by adequately trained medical personnel are fundamental to preventing adverse pediatric outcomes [1]. Australian hospitals have implemented system-level interventions, including universal observation charts, electronic medical records, and patient deterioration guidelines, to support nurses in recognizing pediatric patients’ deterioration [2]. Junior medical doctors are often the first responders to deterioration notification; however, they may lack experience in deterioration management, and most adverse outcomes are caused by missing or misinterpreted patient information. Thus, junior doctors require supervision in acute settings to prevent adverse incidents [3].

Communication failures are frequent causes of adverse events in a pediatric care [4, 5]. Effective communication of the patient’s condition is often lacking among team members, and the transfer of information frequently falls short in conveying clinical information or goals [6]. Currently, junior medical doctors in Australia receive a group orientation session followed by sporadic training at work. They do not receive targeted, individualized feedback sessions before entering the clinical environment, and there is no existing system in place for the early identification of poor performers.

The use of standardized handover instruments improves the transfer of information between clinicians and improves patient outcomes [7]. One of the most well-studied and well-utilised instruments is the ISBAR (Introduction, Situation, Background, Assessment, and Recommendation), which is endorsed by the World Health Organisation and based on the SBAR. In Australia, medical personnel are encouraged to use the ISBAR with all handovers [2, 8, 9]. However, providing an ISBAR form to trainees without formal educational sessions on using the tool does not ensure their competency in communicating critical information [8]. Previous interventional studies focused on group and individual didactic educational sessions using communication tools and reported mixed outcomes [10–12]. Thus, empirical evidence remains limited concerning the most effective method of teaching appropriate communication tools to allow effective escalation of care. A constructivist approach, including applying knowledge, skills, and behaviors to novel situations, is required to develop the complex skill of synthesizing critical information in a wide variety of scenarios.

Simulation is a valuable educational modality, offering an approach for exploring complex and less common medical scenarios, as well as the dynamic interactions among medical personnel in these situations, all without compromising patient safety [13]. These studies reveal a prevalent issue of suboptimal communication, including the dissemination of erroneous information, during medical emergencies. The findings underscore the critical need for enhancing communication training in healthcare settings. Moreover, the efficacy of medical simulation extends beyond crisis scenarios, demonstrating its utility in diverse situations for performance training and evaluation.

To the best of our knowledge, no existing study has combined an individual didactic session with an individualized constructive feedback session to improve an individual’s communication regarding escalation of care. Therefore, the primary aim of this simulation-based study was to assess the influence of an individualized communication training session (a one-on-one didactic session based on the ISBAR communication technique followed by individualized constructive feedback on participant’s performance) on the improvement in the escalation of care in simulated pediatric emergencies. We hypothesize that individualized training sessions will improve completion of elements of the ISBAR, improve the conversation duration, and improve the global rating score achieved by participants. The secondary study aim was to assess the trainees evaluation of the utility of training.

## Materials and methods

The prospective mixed-method pre-post interventional study was conducted over six months (September 2019–February 2020) at Mackay Base Hospital, a regional teaching hospital in Queensland, Australia. Participants included medical students and junior medical officers, including first-year hospital medical officers, second- or third-year post-graduate residents, fourth-year and higher post-graduate principal house officers, and registrars (doctors in an accredited training program). All eligible doctors meeting this criteria were invited to participate in the study, and all doctors invited consented to participate. Written informed consent to participate was obtained from the participants prior to commencement of the study. This study was approved by the Townsville University Hospital Human Ethics Research Committee (HREC/2019/QTHS/6007).

**Fig. 1:**
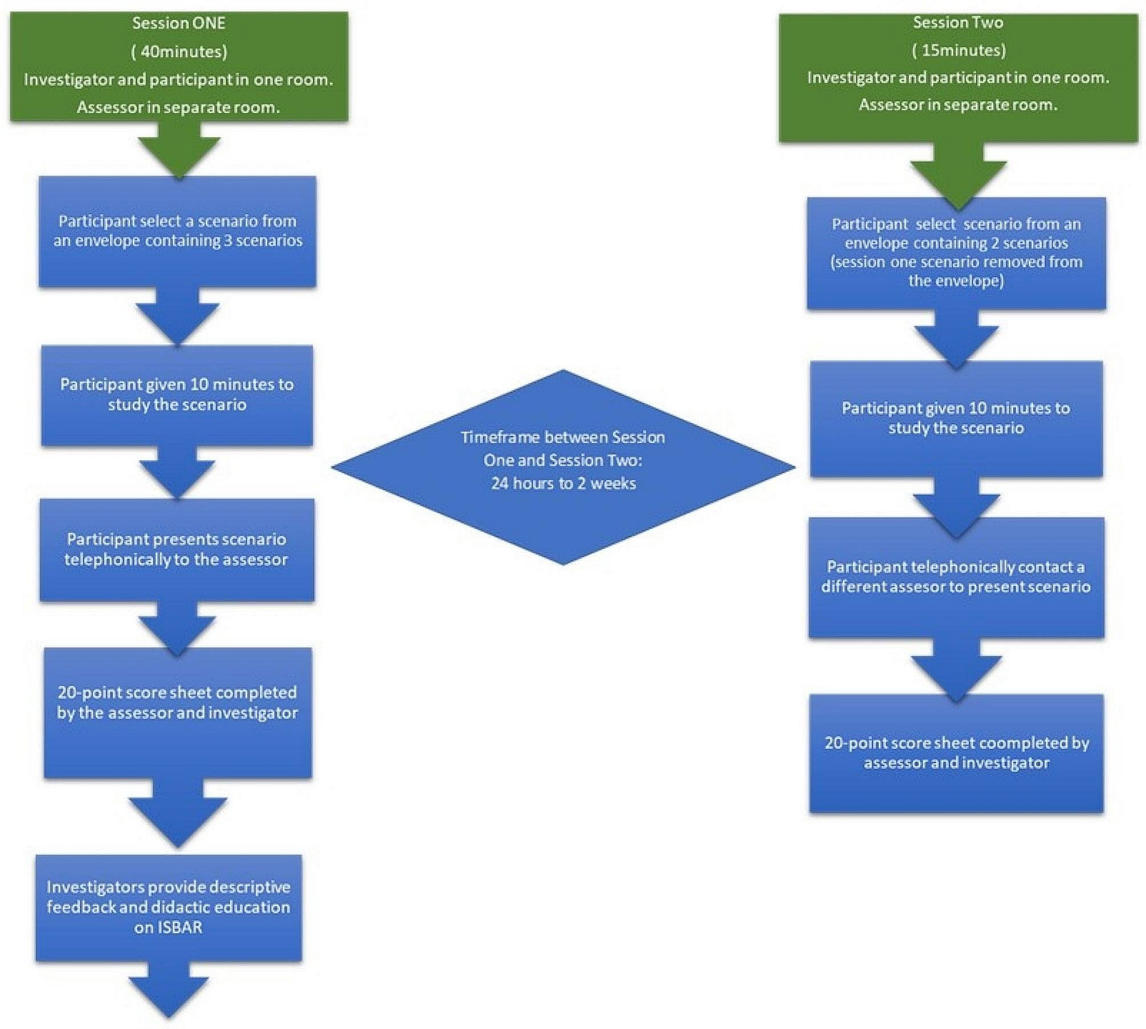
Flow diagram detailing the study method

We conducted the research over two separate sessions to assess participants’ abilities to escalate care in a simulated pediatric emergency scenario before and after the intervention (Fig. 1). Three common pediatric emergency scenarios were chosen for assessment: an 18-month-old child with sepsis, a 5-year-old child with severe respiratory distress, and a 20-month-old child with suspected non-accidental injury (see the Supplementary Table 1 for full details of the scenarios). In all three scenarios, patients exhibited a period with stable vital signs followed by a sudden deterioration. These detailed, unstructured written scenarios were reviewed and approved for complexity by two pediatric consultants.

Each assessment was undertaken by the lead author and one of two pediatric consultants who were randomly assigned to either the first or the second case presentation and were blinded to the intervention status of the participant. To ensure inter-rater reliability the lead author and pediatric consultants undertook a training session on the use of the assessment tool and referred to a standardized check list to direct their ratings. To maintain confidentiality, the participants were deidentified using code names for communications with assessing pediatric consultants.

In Session One, participants selected at random one of the three written case scenarios from an envelope. The participant studied the scenario for up to 10 min and presented it telephonically to a pediatric consultant located in a remote location while the lead author was in the room with the participant. The phone conversation was on speaker so that the lead author could hear the entire conversation. The pediatric consultant and lead author utilized an 18-item score sheet to identify the presence or absence of each component of the ISBAR tool (Table 1), record the duration of the conversation, and provide a global rating score. This ISBAR checklist was the main outcome variable of the study. The overall communication was rated using a 5-point global rating scale (Table 2). The feedback form completed by the pediatric consultants also included “free text” response options. The evaluation tool was adapted from a previous study, in which it was formally used for final-year medical students’ end-of-year assessments [11]. The assessment was performed in real time as the information was being presented via telephone. The consultants were allowed to ask open-ended clarifying questions during the assessment.

**Table 1:** ISBAR checklist with global rating score for overall communication for assessors

I	1. Identify self-position		
	2. Identify self-location		
	3. Identify Receiver name		
	4. Identify Receiver position		
	5. Identify patient name		
	6. Identify patient age		
	7. Identify patient sex		
	8. Identify patient location		
S	9. States early the purpose of the call		
	10. States purpose clearly and concisely		
	11. States if urgent or not		
B	12. States relevant issues in logical order		
	13. States relevant vital signs		
A	14. States the possible diagnosis		
	15. States differential diagnosis		
	16. States if deteriorating or stable		
R	17. Asks for help or advice clearly		
	18. Clarify instructions		
	19. Duration of conversation		
	20. Global rating scale		
Reason for allocated global rating scale score			

Note. ISBAR (Identification, Situation, Background, Assessment, Recommendation)

**Table 2:** Global rating score for overall communication

Marks	Criteria
**1**	Requires frequent prompting, no economy of words, speaks too quickly or rarely pauses, not structured or clear. Uncertain urgency.
**2**	Requires significant prompting, recognises important aspects but includes irrelevant information. Urgency was unclear.
**3**	Some prompting required, concise at times, hesitant or too rapid periodically. Some logical order.
**4**	Occasional prompting needed. Appropriate pace and pauses. Understanding of patient state, urgency, and likely diagnosis. Some confusion about action required.
**5**	Reasoned, coherent, and concise delivery. No prompting needed. Pauses appropriately and clarifies (closes the loop).

Immediately following the case presentation, all participants completed an individualized one-on-one educational session with the lead author, including self-reflection, didactic teaching, and constructive feedback based on the standardized communication tool, over a 30-minute period. During the educational session the lead author used both sets of scoring sheets to inform teaching content. During this session participants were informed of the items they did not complete, the duration of the telephone conversation, and their global rating scale.

**Fig. 2:**
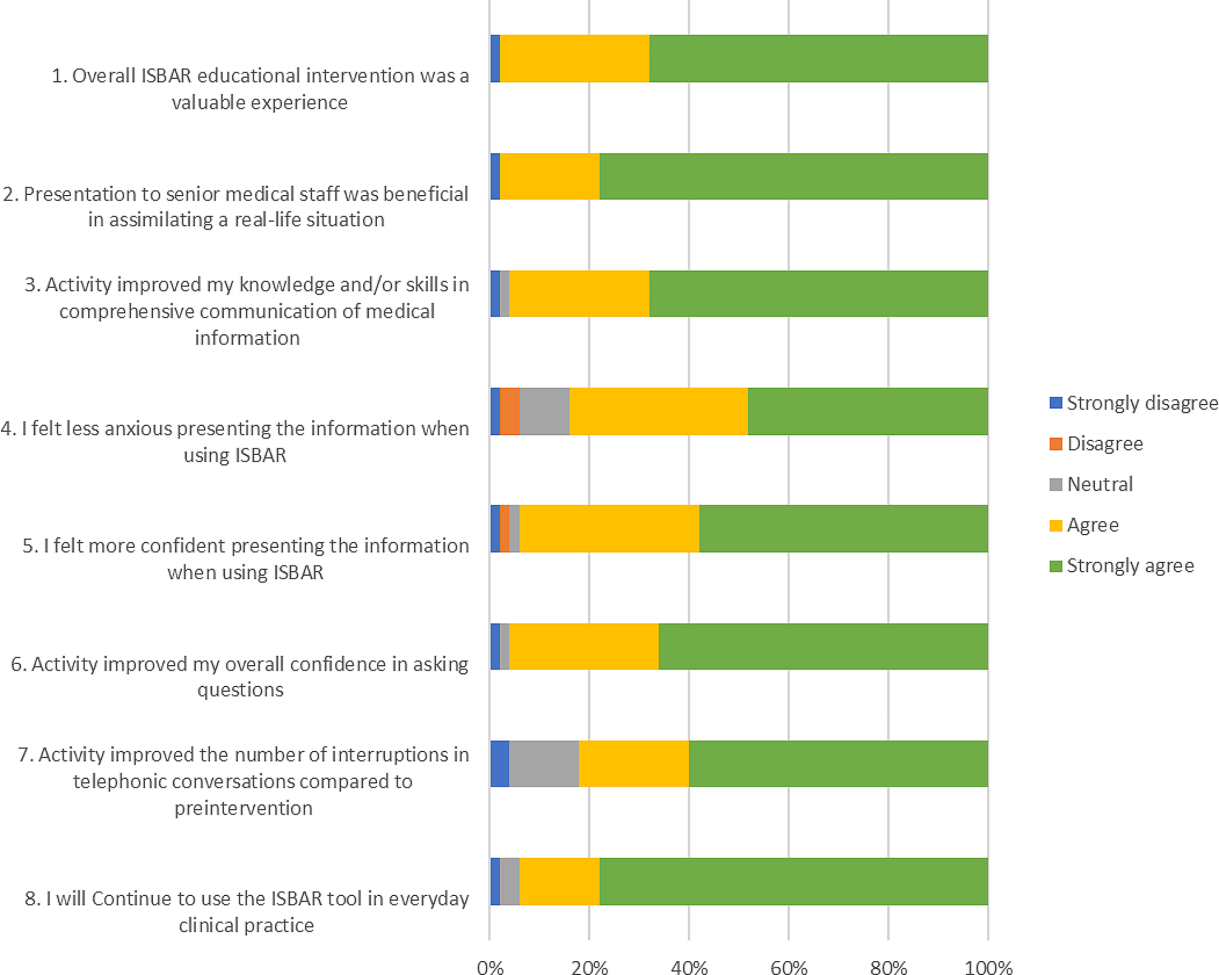
Likert scale responses to question 1–8

In Session Two, participants selected at random from an envelope one of the two remaining scenarios. The participant then undertook a second case presentation and assessment in a similar format, between 24 h to two weeks after Session One. The duration varied between the two sessions to accommodate the logistics of all participants completing their study during the rostered work hours. Participants completed a Likert-type feedback form with “free text” options at the end of Session Two (Fig. 2). In addition to the scoring tools, we undertook qualitative analysis of the pediatric consultants perceptions of the participant’s handover before and after the intervention, and the participant feedback on the training.

### Data analysis

#### Quantitative data analysis

Statistical analysis of the quantitative data was conducted using SPSS version 25.0 (IBM, Chicago, IL, USA) and R software version 3.6.2 (R Core Team, 2019). Categorical data are presented using frequencies and percentages; we analyzed numerical data using descriptive statistics (mean or median values). The differences between pre- and post-intervention scores for each item of the ISBAR checklist and the duration of conversation were analyzed using the McNemar’s test. The Global rating scale was assessed using Wilcoxon signed-rank test. All statistical tests were two-tailed; 95% confidence intervals were reported, and *P* < 0.05 was considered significant.

#### Qualitative data analysis

Qualitative data were analyzed with NVivo 12 (QSR International Pty Ltd. Vic, Australia), which was used to analyze codes and track themes from the pediatric consultants’ and participants’ free-text responses. For this purpose, the conventional content analysis approach [14] was adopted to analyse the qualitative data. The content analysis is appropriate for analysing semi-structured interviews and attempts to elicit participants’ views on their lived experiences [15]. The data was analysed by AK and TE following [16] four-stage thematic analysis: (i) decontextualization, (ii) recontextualization, (iii) categorization, and (iv) compilation. First the decontextualization of the data was done through reading of the text and breaking the text into meaningful units and groups. The second state, recontextualization was achieved through re-reading the original text alongside the final list of meaning units, reviewing, and identifying unrelated texts, and excluding them from the analysis. In the third stage, categorization of the units was done to derived themes and sub-themes from the data. These themes were discussed between the lead author and pediatric consultants until consensus was reached and were later shared with the entire research team for discussions and approval to represent the meanings they conveyed in the data. Finally, the themes were compiled and verbatim quotes were used to represent participants experiences in the main report (Bengtsson, 2016).

## Results

The study participants included 50 junior medical officers (eight medical students, 31 residents, eight registrars, and three principal house officers), including 33 (66%) female doctors and 17 (34%) male doctors. A total of 19 (38%) participants had worked in a general pediatric inpatient unit within the last year, and nine (18%) had over five years of professional experience.

Post-intervention, significant improvement was noted in eight of the 18 components of the ISBAR checklist, as summarized in Table 3. The ‘identification’ component of ISBAR was the only domain where no components improved following individualized communication training, reflecting that participants performed well pre- and post-intervention in all eight components of this domain.

**Table 3:** ISBAR checklist data showing areas with statistically significant improvement

Variable	Pre N (%)	Post N (%)	P value
Identify self-position	49 (98)	48 (96)	1.000
Identify self-location	50 (100)	49 (98)	-
Identify Receiver name	34 (68)	41 (82)	0.143
Identify Receiver position	49 (98)	48 (96)	1.000
Identify patient name	49 (98)	42 (84)	0.581
Identify patient age	49 (98)	47 (94)	0.625
Identify patient sex	46 (92)	46 (92)	1.000
Identify patient location	41 (82)	47 (94)	0.146
States the purpose of the call early	29 (58)	44 (88)	0.002
States the purpose clearly and concisely	25 (50)	44 (88)	< 0.001
States whether urgent or not	21 (42)	43 (86)	< 0.001
States relevant issues in logical order	28 (56)	45 (90)	< 0.001
States relevant vital signs	38 (76)	47 (94)	0.030
States the possible diagnosis	46 (92)	50 (100)	-
States the differential diagnosis	10 [17]	16 (32)	0.263
States if deteriorating or stable	34 (68)	46 (92)	0.004
Asks for help or advice clearly	32 (64)	46 (92)	< 0.001
Clarifies instructions	19 (38)	40 (80)	< 0.001

Note: ISBAR: Identification, Situation, Background, Assessment, Recommendation

We also did not see any significant improvement in participants’ abilities to explore differential diagnoses (*P* = 0.263). Notably, most participants performed poorly both pre- and post-intervention in this area. However, pediatric trainees were more likely to present differential diagnoses since five of the six of them presented good overall communication and were able to provide differential diagnoses pre-intervention.

**Fig. 3:**
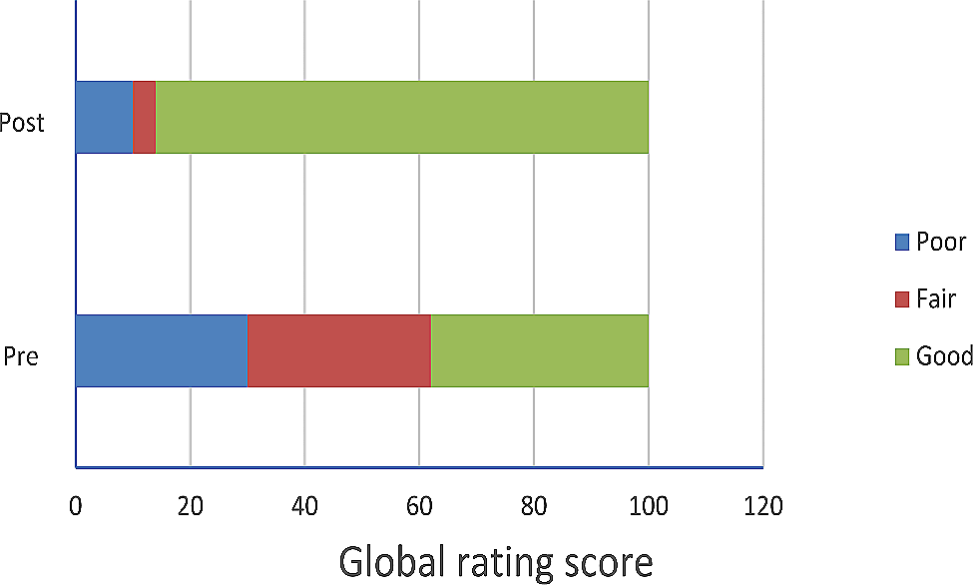
Comparison of pre- and post-intervention global communication scores: Good (score 4 or 5), Fair (score 3), Poor (score 0–2)

Escalation of care involves four components of the ISBAR that were significantly improved post-intervention– states if the call was urgent (*P < 0.001*); states relevant vital signs (*P = 0.03*); states if deteriorating or stable (*P = 0.004*); and asks for help or advice clearly (*P* < 0.001). Further analysis showed that this improvement was associated with improvement in overall communication as measured by the global rating scale (Fig. 3, *P* < 0.001).

Participant feedback suggested overall improvement in self-confidence when presenting post-intervention with the ISBAR format (Fig. 2). In addition, most of the participants (98%) reported that individualized communication training was a valuable experience (Fig. 2). Overall, 34 (68%) participants strongly agreed that the intervention improved their communication skills (Fig. 2).

A total of 13 pre-intervention and 14 post-intervention pediatric consultant feedback forms and 32 participant feedback forms were returned without any free text response. Using data from the remaining 73 and 18 responses, respectively, authors identified thematic categories for each stage. The post-intervention main themes were [1] “improved urgency for assistance from senior doctors” and [2] ”conversational clarity”.

The two major themes from the participants’ descriptions were [1] intervention quality (e.g., “I think it is helpful for people starting up. A video presentation of a clinical scenario may be something to look at in the future to make the scenario closer to reality”), and [2] confidence (e.g., “I feel more confident presenting to a consultant with the ISBAR format”).

## Discussion

Our study aimed to evaluate the effectiveness of individualized one-on-one communication training for junior medical doctors in the context of pediatric emergencies. Our findings reveal substantial improvements in several components of the ISBAR checklist, enhancing overall communication during care escalation. These insights underscore the importance of tailored training in optimizing emergency communication.

Previous simulation-based studies have examined group and individual didactic training regarding communication tools among medical, nursing, pharmacy students, and junior medical doctors [10–12]. The outcomes of these studies indicate that training in the use of ISBAR is both feasible and effective. For example, Marshall and colleagues demonstrated that such training is likely to yield improvements in communication within the clinical environment, particularly among junior clinicians when making telephone referrals [11]. These findings underscore the potential impact of targeted educational interventions in enhancing communication skills among healthcare professionals. However, previous studies with junior medical doctors have reported mixed results regarding the best mode for providing communication skills education [10–12].

A randomized control study with interns by Cunningham et al. showed no improvement in telephonic transfer of critical data post-exposure to a 10-minute one-on-one didactic training session on SBAR [10]. It was hypothesized that telephone referral skills are influenced by a combination of factors, including problem recognition, feedback, and the availability of targeted skill teaching using SBAR or other communication strategies [10]. In this study, we combined a didactic communication training session with self-evaluation and feedback and found a positive effect on the transfer of critical data confirmed by significant improvement in eight out of 18 components of the ISBAR checklist. Despite some similarities between the two studies, it is important to note the significant differences in methodology and participant skill level between the previous study and the present study. The study by Cunningham and colleagues was limited to a 10-minute didactic educational intervention with an “emotion-centered” debriefing, not focused on clinical or performance issues. In contrast, the present study implemented a longer intervention (30 min) focused on self-evaluation and feedback regarding participants’ performance.

However, problem recognition, as identified in previous studies, is a skill that requires “higher-order thinking” and “experience” [10, 18]. Our results demonstrated that, despite having a format to consider different possibilities for the clinical presentation, there was no statistical improvement in participants’ ability to state differential diagnoses. Participants with experience in pediatric presentations were more likely to suggest differential diagnoses and management options. Thus, it may be that junior medical doctors require more pediatric-focused simulations, real-life bedside teaching, and pediatric experience to present differential diagnoses. Theoretically, the diagnostic process improves the understanding of patient management and prognosis. Thus, improving care management for deteriorating patients requires critical data transfer; it also requires junior doctors to be able to clearly communicate their needs for urgent senior staff supervision.

The reasons for junior medical doctors’ failure to escalate their concerns about a deteriorating patient are often complex. Callaghan and colleagues undertook a previous integrative review of 33 articles exploring junior medical doctors’ skills in managing critically ill or deteriorating patients, indicating there is substantial room for improvement in junior medical doctors’ capacity to manage these situations [3]. Increased senior supervision and training in communication skills for junior medical doctors have been hypothesized as essential; thus, improving junior doctors’ ability to escalate care is important in managing deteriorating patients [3]. We found that the mean performance for escalation of care for both medical students and junior medical doctors were significantly higher in the simulated environment after the one-on-one individualized, constructive feedback communication training intervention. Future studies should determine if this result can be replicated in the clinical environment. Additional research is also needed to explore ways to support poor performers, such as with repeated interventions.

Additionally, communication is one of the most common “procedures” practiced in medicine [19], and conversational clarity when discussing a deteriorating pediatric patient is much more challenging without visual and nonverbal cues [20]. Our study identified individual areas in which the participants struggled, and support with focused assistance was effective in improving conversational clarity in a simulated setting. Moreover, we evaluated participants’ self-confidence post-intervention. Consistent with previous studies, our participants reported feeling more confident with referrals post-intervention [17].

Our study has several strengths. (1) Unlike previous one-on-one communication studies with brief didactic teaching, this study combined teaching with self-evaluation and individualized feedback. (2) This was a mixed-method study, which generated evidence regarding the effectiveness of the training. (3) The participant drop-out rate was zero. (4) The participants were deidentified, and the pediatric consultants were unaware of whether the participant was presenting pre- or post-intervention. (5) We recruited a sample of junior medical officers with diverse experience levels in terms of specialty and exposure to the Australian healthcare system.

Despite these strengths, our study had several limitations. The study comprised a heterogeneous group of junior medical doctors; thus, we could not obtain standardized baselines prior to the commencement of the study, and it was difficult to generalize the results to a specific year of training. Generalization is easier with a homogeneous population but recruiting a homogeneous population is difficult in practice because trainees come from different backgrounds and have different levels of training; some also possess overseas experience. The findings represent a single institution, and caution is needed when generalizing how they apply to other services. Although participants were deidentified, some of the junior doctors were well known to the pediatric consultants and likely identified via voice recognition. All participants attended two separate sessions, which were a minimum of 24 h and a maximum of two weeks apart. This limitation may be due to the time constraints and logistical constraints that also prevented us from conducting a longitudinal study with ongoing serial measurements to assess decay in retained knowledge.

## Conclusions

Our study reported that individualized, targeted communication training (didactic with self-evaluation and feedback) for junior medical doctors was beneficial for improving pediatric escalation of care, overall communication, and junior medical doctors’ confidence level within a simulated environment. Thus, institutions should consider individualized communication training with targeted constructive feedback from senior medical officers to improve communication about the escalation of care.

### Next steps

Future studies should explore the use of this model with various training levels, examined separately, to assess the full benefits of the intervention and determine if positive findings are sustained over the longer term. Furthermore, studies should explore the impact of such targeted interventions on regular clinic work and their effects on reducing adverse clinical events.

### Electronic supplementary material

Below is the link to the electronic supplementary material.

Supplementary Material 1

## Data Availability

Data pertaining to the results of the study is available from the corresponding author upon request.
