# Supplementary material for: Descriptive feedback with targeted education to improve telephonic escalation of care: a simulation-based study
Source: BMC Med Educ. 2024 Mar 13;24:281. doi: 10.1186/s12909-024-05260-1 (PMC10938749; doi:10.1186/s12909-024-05260-1)
Supplement: Supplementary file 1 — Supplementary Material 1 [file 12909_2024_5260_MOESM1_ESM.docx]

**Scenario 1: April 18-month-old girl with possible septic shock**

**April is seen with mother Julie.**

History:

- April has been unwell for one day.
- She had minimal intake over the last 24 hours.
- April had a temperature of 39.3 degrees Celsius on arrival to triage.
- April had no runny nose, but coughed a few times followed by one milky vomit.
- She passed a normal bowel motion in the morning.
- She attends day-care 3 days per week.
- No known specific unwell contacts reported in day- care or at home.
- Immunization are all up to date.
- She is not known with any specific allergies.

Examination Findings:

- Vitals: Heart rate: 160/min, afebrile, Respiratory rate: 26breaths/min, oxygen saturation: 98% in room air, clinically looked mottled.
- April was clingy with mother and resisting examination.
- April has no rash and had a clear chest with no work of breathing.
- Her fontanelle are closed with a normal abdominal examination.
- Small cervical lymph nodes palpable bilaterally.
- Lips dry but mucous membrane was moist.
- ENT exam was normal with clear Tympanic membrane and normal-sized tonsils.
- On exam she was Grunting and had a capillary refill time: 2-3 sec initially which prolonged to 3 sec centrally and 5 sec peripherally.
- Laboratory findings: Haemoglobin: 120, CRP: 129, blood culture negative, WCC: 17.6, neutrophil: 14.4, urine pending.

Interventions done:

- Initiated on high-flow nasal cannula and fluid bolus.
- Administered cefotaxime.

Follow up considerations.

- Requesting consultant review urgently.
- Considering admission to ward vs ICU based on clinical deterioration.
- Pending further investigations and antibiotic coverage.

**Scenario 2: Jamie, 20-month-old boy with head injury**

**Accompanied with mother Sally**

History:

- Sally reported that Jamie fell from his bed, unwitnessed, while playing with his older brother.
- Sally heard a loud bang followed by Jamie screaming.
- Sally found Jamie lying quietly on the floor and he appeared lethargic and poorly responsive.
- After 4-5 min Jamie started crying and responding appropriately.
- Jamie vomited once in the car on the way to the hospital.
- He has been Immunized up to date and has no known allergies.
- He was born at term via normal vaginal delivery, with one admission for bronchiolitis in the past.
- Parents are married and currently unemployed.

Examination Findings:

- Jamie was awake but pale, lying quietly on the bed.
- Jamie had a left parietal scalp hematoma measuring 4cm.
- He was cooperative but dazed and not verbalizing.
- He had few linear bruises over right buttock and right ear.
- Pupils were equal and reactive.
- His weight was on the 3rd percentile, head circumference on 75th percentile, height on 50th percentile.

Follow-up Considerations:

- Considering urgent CT brain for assessment of intracranial injury.
- Contemplating admission for observation.
- Planning to organize child protection services and involve social worker.
- Intending to perform blood tests including coagulation profile and full blood count.

Scenario 3

**Scenario: Emily, 6-year-old girl with suspected asthma exacerbation**

History:

- Emily presents with a history of wheezing and shortness of breath for the past 24 hours.
- Her symptoms worsened overnight, with increased coughing and difficulty breathing.
- She has a past medical history of asthma, with occasional exacerbations managed at home with a reliever inhaler.
- No recent illnesses or known triggers for her asthma exacerbation.
- No known allergies to medications.
- Lives in a smoke-free environment.
- Immunizations up to date.

Examination Findings:

- Emily appears anxious and uncomfortable, sitting upright and using accessory muscles to breathe.
- Respiratory rate: 30breaths/min, heart rate: 110/min, oxygen saturation: 92% on room air which further worsened to RR 55 breaths/minute, Heart rate 150/min and saturations of 91% on room air.
- Auscultation reveals widespread wheezing bilaterally.
- Chest is mildly hyperinflated.
- No signs of respiratory distress at rest.
- No cyanosis or altered mental status.
- No fever or signs of infection.

Interventions done.

- Administered initial burst of Ventolin and oral prednisolone.
- Monitored response to treatment closely, with repeated assessments of respiratory rate, heart rate, and oxygen saturation.
- Initiated supplemental oxygen therapy to maintain oxygen saturation > 94%.

Follow-up Considerations:

- Minimum improvement in work of breathing and tachypnoea.
- Urgent Consultant review and discussion with ICU
- Consider IV canula with IV steroids, CXR and IV antibiotics
